# Comparison of Polydrug Use Prevalences and Typologies between Men Who Have Sex with Men and General Population Men, in Madrid and Barcelona

**DOI:** 10.3390/ijerph182111609

**Published:** 2021-11-04

**Authors:** Juan-Miguel Guerras, Juan Hoyos, Patricia García de Olalla, Luis de la Fuente, Lidia Herrero, David Palma, Jorge del Romero, Jorge-Néstor García-Pérez, María-José Belza, The Methysos Project Group

**Affiliations:** 1 Centro Nacional de Epidemiología, Instituto de Salud Carlos III, 28029 Madrid, Spain; jguerras@isciii.es (J.-M.G.); lfuente@isciii.es (L.d.l.F.); lidia.herrero.huertas@gmail.com (L.H.); 2 CIBER Epidemiología y Salud Pública (CIBERESP), 28029 Madrid, Spain; polalla@aspb.cat (P.G.d.O.); ext_dpalma@aspb.cat (D.P.); mbelza@isciii.es (M.-J.B.); 3 Departamento de Salud Pública y Materno-Infantil, Universidad Complutense de Madrid, 28040 Madrid, Spain; 4 Servicio de Epidemiología, Agència de Salut Pública de Barcelona, 08023 Barcelona, Spain; 5 Centro Sanitario Sandoval, Instituto de Investigación Sanitaria San Carlos, Hospital Clínico San Carlos, 28010 Madrid, Spain; jromero@salud.madrid.org; 6 Unidad de ITS de Vall d’Hebron-Drassanes, Hospital Vall d’Hebron, 08001 Barcelona, Spain; g.perez@vhebron.net; 7 Escuela Nacional de Sanidad, Instituto de Salud Carlos III, 28029 Madrid, Spain

**Keywords:** men who have sex with men, heterosexual men, drug use, polydrug use, comparison

## Abstract

This study compares the prevalence of drug use and the typologies of polydrug use (PDU) in men who have sex with men (MSM) and general population men (GPM). Participants were men aged 16–64, living in the provinces of Madrid and Barcelona: 1720 were recruited in a GPM survey, and 2658 were HIV-negative MSM from HIV/STIs diagnosis services. Lifetime and last-year prevalence of drug use and prevalence ratios (PRs) of MSM to GPM for the different drugs were calculated using Poisson regression. Latent class analysis (LCA) was performed to identify typologies of PDU. Lifetime use of the drugs considered was higher in MSM, and even higher for drug use in the last-year: PRs for cannabis, hallucinogens and cocaine ranged from 2–5; for amphetamine, ecstasy and methamphetamine 12–16; and above 60 for ketamine, GHB/GBL, inhalants and mephedrone. In the LCA for lifetime PDU four classes arose from the GPM (No-PDU (79.6%); Conventional PDU (13.8%); Intensive conventional PDU (4.9%); Heavy PDU (1.8%)) and four among MSM (No-PDU (57.7%); Conventional PDU plus poppers (18.8%); PDU preferring chemsex drugs (6.4%); Heavy PDU (17.2%)). For PDU during the last-year, three classes arose in the GPM: No-PDU (94.7%); Conventional PDU (4.3%); Heavy PDU (0.9%). For MSM, we identified four classes: No-PDU (64.7%); Conventional PDU plus poppers (15.6%); PDU preferring chemsex drugs (6.2%); Heavy PDU (13.5%). MSM should be considered a priority group for the prevention of the use of all drugs but the heterogeneity of PDU typologies regarding users’ preference towards conventional and/or sexualised drugs needs to be taken into account.

## 1. Introduction

Men who have sex with men (MSM) usually show poorer health indicators than exclusively heterosexual men (HM) or general population men (GPM, that includes both MSM and HM), including mental disorders (e.g., depression or anxiety), chronic diseases or disabilities, and higher prevalence of human immunodeficiency virus (HIV), hepatitis C virus (HCV) and other blood-borne or sexually transmitted infections (STIs) [1,2,3,4,5,6]. Although less family and social support, discrimination, stigma, and victimization, as well as greater barriers to accessing optimal health care across their life spans, could contribute to this health disadvantage, it is probable that the greater frequency of particular unhealthy behaviours such as drug use, including tobacco and alcohol, or high-risk sexual practices are very important contributory factors to these health inequalities [1,4,7].

During the last decade a large number of research papers has been published, which have focused on the use of different drugs immediately before or during sex (sexualised drug use, SDU), especially centred on certain drugs whose use has spread recently among MSM and that have been commonly labelled as “chem drugs”: crystal methamphetamine, gamma-hydroxybutyrate/gamma-hydroxybutirolactone (GHB/GBL), mephedrone and, sometimes, ketamine. However, all these studies show that the prevalences of SDU of more traditional drugs (such as cannabis, cocaine, ecstasy or poppers) tend to be much higher than the so called “chem drugs” [8,9,10,11,12,13,14]. That said, while a very important factor, sex is only one of the reasons for using drugs, there are many others, and—with the exception of alkyl nitrites and the four chem drugs—psychoactive drugs are probably used more frequently by most MSM for purposes other than sex. In addition, some recent articles suggest that drug use, both for sex and recreational purposes, is increasing, in both HIV-positive [15] and HIV-negative MSM [16].

It is always assumed that the prevalence of drug use is much higher in MSM than in their heterosexual counterparts, although there are not many studies outside the USA comparing prevalence-of-use or related problems. First, it is not uncommon for authors to claim in their conclusions that MSM have higher levels of drug use than their heterosexual counterparts while having only MSM populations in their studies and no heterosexual men [17,18,19]. So these conclusions are only based on a rough direct comparison of their findings with the global figures for all males included in general population surveys, without any consideration of age structure differences. Second, many of these studies are restricted to alcohol, tobacco, cannabis or illicit/recreational drugs taken together, without considering the specific drugs involved [4,20,21,22,23,24,25]. All of these latter studies are based exclusively on general population surveys where the percentage of men who report having sex with other men is very low, making it very difficult to compare the prevalence-of-use of specific illicit drugs between MSM and other men. Practically all of these studies come from the USA except one from the UK [4], one from Australia [26] and another from Sweden [22] and these three have been published since 2016, because previous population surveys did not include questions about sexual behaviour. As far as we know, only four studies that actually compare particular levels of disaggregated prevalences of different drugs have been published [27,28,29,30]. One [28] was carried out among men (MSM and HM) attending two sexual health clinics in the UK, although this was in a fairly limited sample and did not include age adjustment. The other three studies come from the USA and present data for groups of drugs, but do not provide separate information for some specific substances of great interest: ecstasy, methamphetamine, ketamine, GHB/GBL and mephedrone [27,29,30].

On the other hand, in recent years there has been a growing interest in studying polydrug use (PDU) to classify users into different profiles or typologies (two or more subgroups with meaningful homogeneous characteristics) which take advantage of new methodologies, especially Latent Class Analysis (LCA). This methodology offers substantial advantages compared with more traditional techniques such as Cluster Analysis. LCA has been described as a person-oriented approach, because it focus on similarities and differences among individuals instead of relations among variables [31]. This approach has been used more frequently to identify subgroups of SDU among MSM, but its use in the analysis of PDU for any purpose is very scarce [32,33,34,35]. None of the aforementioned studies that have compared the prevalence between MSM and HM have performed LCA in the two populations to analyse how similar the subgroups of polydrug users and their prevalences are in the different populations.

The areas of Madrid and Barcelona are the two most populated in Spain and have higher prevalences of MSM [13,36,37]. Previous studies have shown that they are among the European cities with the highest SDU prevalences [8,9]. However, no study in these cities, nor in Spain, has ever compared drug-use prevalence for any purpose between MSM and GPM.

In this context, the objectives of this study were: first, to compare drug-use prevalence between a wide sample of HIV-negative MSM attending HIV/STI services in Madrid and Barcelona, and GPM (as a proxy of HM) from a general population survey; second, to identify the main subgroups of polydrug users in the two populations.

## 2. Materials and Methods

### 2.1. Study Design and Participants

This is a cross-sectional study, which is part of the Methysos research project whose purpose is to assess drug use (including SDU) in MSM. Participants were: (a) Subpopulation of MSM: 2658 men, with the following inclusion criteria: aged 16–64, living in the provinces of Madrid and Barcelona, attending early HIV or STI diagnosis services, self-reported having previously had anal sex with other men and never having been diagnosed with an HIV infection. They were recruited in 2018–2020 across four facilities, the two most important STI clinics in Spain (Sandoval in Madrid and Drassanes in Barcelona) and two community programmes for rapid HIV-testing (Pink Peace in Madrid and Agencia de Salut Pública in Barcelona). The STI clinics are basically on demand services and perform testing for all STIs. The community programmes also carry out active recruitment, including via the use of dating apps or websites for MSM and only offer rapid testing for HIV, syphilis and, during this project, also for HCV. (b) Subpopulation of GPM: 1720 men, aged 16–64 and living in the same two provinces. GPM were representative of general-population men and recruited in 2018 within the framework of the EDADES Survey which uses three-stage random sampling (census sections, households and individuals). More details can be seen in the report of that survey [38]. The sample was stratified by age and region of residence.

### 2.2. Variables and Data Collection

In addition to questions on drug use, the two surveys have some sociodemographic variables in common: age, country of birth, province of residence, size of town of residence, highest education level achieved and employment status. The two surveys asked about the use for any purpose of the different drugs considered in the current original: cannabis, hallucinogens, cocaine, amphetamine, ecstasy, methamphetamine, ketamine, GHB/GBL, volatile inhalants (poppers) and mephedrone. They also asked about the use of another two groups of drugs: sedatives/tranquilizers/hypnotics and opioids. However, the EDADES survey explicitly distinguished between its use “with” and “without” prescription, while the MSM survey did not include this distinction. The wording of the MSM study was tilted towards illegal use, because the word “drogas” in Spanish does not usually include legally prescribed medication. In the case of opioids, EDADES asked separately about heroin (self-reported) and many questions (by interview) about the use of many specific prescribed opioid analgesics. However, the MSM survey asked about the use of heroin and other opioids in a similar way to that of tranquilizers. Due to these differences, these two groups of drugs were considered as not comparable and were thus not included in the analysis. Detailed definitions of drug-use variables in the two questionnaires can be found in Supplementary Table S1.

GPM data were collected through a paper-and-pencil self-administered questionnaire (drug-use questions) or a face-to-face interview (socio-demographic questions) [38] and MSM data through an online self-administered questionnaire, without personal identifiers, using a tablet while waiting for consultation in the facilities.

The study was approved by the Research Ethics Committee of the Instituto de Salud Carlos III (CEI PI 44_2018_subproyecto1-v2 and CEI PI 44_2018_subproyecto2).

### 2.3. Data Analysis

Regarding outcomes, lifetime and last-year drug-use prevalence and its corresponding 95% confidence intervals (95% CI) were calculated. We compared the outcomes and independent variables (sociodemographic) between MSM and GPM. Comparisons of independent variables were assessed using Pearson’s χ^2^ or Fisher’s exact tests rejecting the null hypothesis if *p* < 0.05. The relative difference in drug-use prevalence was assessed with the crude prevalence ratio (cPR) of MSM to GPM. The variance of cPR to calculate 95% CI was estimated using the normal approximation of the natural logarithm of cPR. Finally, to compare the prevalence adjusting for other independent covariates the adjusted PR (aPR) and its corresponding 95% CI were estimated from Poisson regression with robust variance in the framework of generalized linear models [39,40]. These analyses were performed using Stata v. 15 (Statacorp, College Station, TX, USA). Although the “age” sampling design implied the use of weights, in the present analysis these weights were not taken into account, as they primarily affected region and age, and here we analysed only two regions and the aPRs were age-adjusted.

Latent class analysis (LCA) [41] was performed to identify and characterize meaningful latent classes or subgroups of participants who have similar typologies of PDU in the reference period. We constructed ten dichotomous variables for lifetime-use and another ten for last-year-use of the following drugs: cannabis, hallucinogens, cocaine, amphetamine, ecstasy, methamphetamine, ketamine, GHB/GBL, volatile inhalants, and mephedrone. We considered two different approaches. The first was to carry out different latent class analyses for MSM and for GPM, and the second to perform one latent class analysis for the entire population and to compare whether the prevalence of these typologies differs between MSM and GPM. Given the very wide range of PRs for the different drugs, we thought that the existence of very different patterns among MSM and GPM were very likely and that the conditional probabilities for the use of each substance would be radically different for each subpopulation. For these reasons we thought it would be more appropriate to perform a stratified analysis, similar to that performed by Achterbergh to compare patterns of drug use for sex [42]. The results of the stratified analysis confirmed our hypotheses, so it did not make much sense to perform the pattern analysis for the entire population. Using the R package “PoLCA” [43] our sets of models were constructed: two for MSM (one for lifetime use and another for last-year-use) and another two for GPM. For each set we tested models with 2–6 classes. For the estimation of each model, a maximum of 20 repetitions with different sets of random starting values (for avoiding under-identification) and 1000 iterations per repetition were fixed. With this procedure all the models with 2–6 classes converged. For the analysis of the goodness of fit of the models with different numbers of classes the following statistical information criteria were calculated: maximum log-likelihood, G2, AIC, CAIC, BIC, ABIC, and relative entropy [44]. The model also provides the probability (prevalence) of each latent class and the “conditional probabilities,” which estimates the probability of a participant using each of the drugs considered, given their membership of that class. In order to select the final model with a specific number of classes, in addition to statistical information criteria of model fit, we also contemplated and balanced other considerations, such as parsimony and interpretability, including the fact that we were comparing two populations.

The proportion of missing values for outcomes ranged between 0.0% (MSM) and 4.3% (last-year cannabis use) in GPM, and the proportions for independent variables were less than 3%, except for area-of-birth among GPM, which was 8.8%. Employment status and cohabitation among MSM were not collected in Barcelona. We performed a sensitivity analysis for Madrid, to explore the effect of employment status and cohabitation on aPRs.

## 3. Results

### 3.1. Participant’s General Characteristics

Most participants were aged 16–34, born in Spain, living in urban areas, with secondary/university education, and in employment. Compared to GPM, MSM were younger, and more likely to be immigrants (born abroad), resident in cities with more than one million inhabitants (Madrid or Barcelona) and secondary/university educated. The MSM immigrants came mainly from Latin America while GPM were predominantly from other countries (Table 1). In addition, immigrants’ length of stay in Spain was considerably lower for MSM than GPM (7.3 vs. 12.6 years, *p* < 0.001).

### 3.2. Prevalence of Drug Use in MSM and GPM

Of the drugs considered, the most widespread were cannabis, cocaine and ecstasy, with the addition of volatile inhalants among MSM (Table 2). Thus, the four drugs with the highest prevalence-of-use in the last-year among MSM were volatile inhalants (51.7%), cannabis (40.7%), cocaine (23.6%) and ecstasy (23.1%), while among GPM these were cannabis (17.8%), cocaine (5.1%), ecstasy (1.8%) and hallucinogens (1.2%). The prevalence-of-use of all the drugs considered was significantly higher in MSM than GPM whether the reference period was over a lifetime or during the previous year.

### 3.3. Differences in Drug Use Risk between the Two Groups of Men

The crude relative difference between MSM and GPM in the extent (prevalence) of drug use in general (any drug) as measured by the cPR was 1.6 for lifetime and 3.4 for last-year. However, the cPR magnitude was very different for each specific drug. Thus, focusing on last-year, the cPRs for cannabis, hallucinogens and cocaine ranged from 2–5, those for amphetamine, ecstasy and methamphetamine from 12–15, and those for ketamine, GHB/GBL, volatile inhalants and mephedrone were over 60 (Table 2).

The adjusted risk of drug-use in general (any drug) as well as the risk of use of each specific drug was significantly higher in MSM than GPM for both lifetime and last-year, except for hallucinogens where the 95% CI for aPR for lifetime included the value 1 (Table 3). The aPR for drug-use risk in general was 1.5 for lifetime and 3.1 for the last-year. However, the magnitude of aPR was very different for each specific drug. Thus, focusing on last-year, the aPR for cannabis, hallucinogens and cocaine ranged from 2–4; those for amphetamine, ecstasy and methamphetamine from 10–14; and that for ketamine, GHB/GBL, volatile inhalants and mephedrone was higher than 70.

In a sensitivity analysis in Madrid, aPRs decreased somewhat when employment status and cohabitation were added to the set of adjustment covariates, although they continued to be very high, especially for drugs other than hallucinogens, cannabis and cocaine (Supplementary Table S2).

### 3.4. PDU Typologies

**Figure 1:**
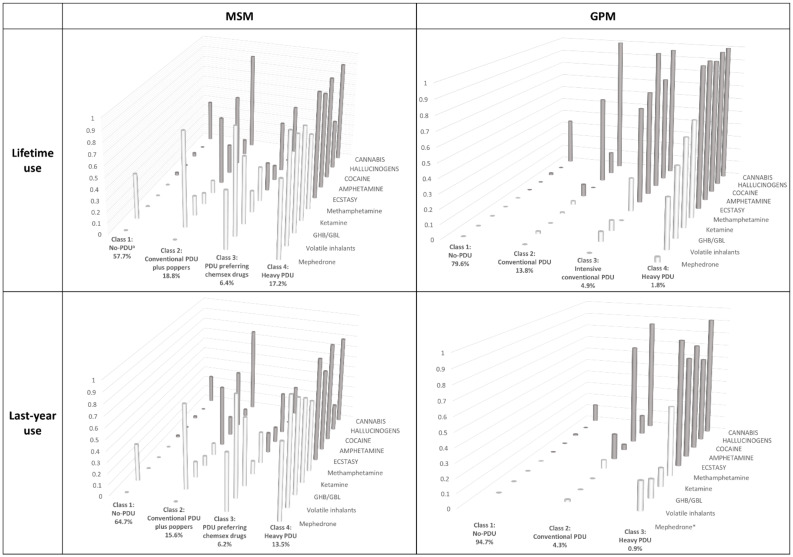
Results of Latent Class Analysis among men who have sex with men (MSM) and general-population men (GPM) for lifetime and last-year use: classes, prevalence of each class and conditional probabilities. ^**a**^ Polydrug use. * Mephedrone is not displayed because there were no reported users for last-year among GPM. White bars: “More traditional drugs”; Black bars: “chemsex drugs”.

Supplementary Table S3 shows the goodness-of-fit statistical information criteria used to select the final models in the LCA. Figure 1 and Supplementary Table S4 display the four final LCA models for MSM and GPM, and for lifetime and last-year prevalence of the ten aforementioned drugs: three LCA with four-class models and only one with a three-class model (last-year use in GPM).

In MSM, focusing on lifetime use, the four latent classes identified were:

- Class (1) No-PDU, with a high probability of only cannabis and volatile inhalants use (0.38 and 0.41) and a very low or zero probability (<0.05) of using any other drugs considered;
- Class (2) Conventional PDU plus poppers, with extremely high probability of using both cannabis and volatile inhalants (0.85), very high probability of using cocaine and ecstasy (>0.6), and medium probability of using amphetamine, methamphetamine and GHB/GBL (0.12–0.27);
- Class (3) PDU preferring chemsex drugs, with almost universal use of volatile inhalants (0.95), very high probability of using GHB/GBL, mephedrone, methamphetamine, cocaine and cannabis (0.44–0.61), and intermediate probability of using other drugs under consideration, except hallucinogens;
- Class (4) Heavy-PDU, with extremely high use-probability for all drugs (≥0.68) except hallucinogens.

The prevalence of Class 1 was 57.7% and that of Class 4, 17.2%.

Focusing on last-year drug-use, the model found the same four latent classes as for lifetime use: however, the conditional probabilities of using any of the drugs were slightly lower in all the classes and the prevalence of the classes increased for No-PDU (64.7%) and decreased for heavy-PDU (13.5%).

For GPM lifetime use, four latent classes were also identified:

- Class (1) No-PDU, very similar to the homonymous MSM class but with hardly any volatile inhalant use;
- Class (2) Conventional PDU, similar to the homonymous MSM group, but with zero or ≤2% probability of inhalant, GHB/GBL, ketamine, mephedrone, amphetamine, and methamphetamine use, lower probability of ecstasy use, and similar use of cocaine, hallucinogens, and cannabis;
- Class (3) Intensive conventional-PDU, shows higher levels of more traditional drugs being used than MSM;
- Class (4) Heavy-PDU, again similar to the MSM group, but with practically universal use of non-chemsex drugs and lower chemsex drug use, especially mephedrone.

The prevalence for Class 1 was 79.6% and Class 4 only 1.8%.

For last-year drug-use, only three latent classes were identified, because that most similar to Class 3 for lifetime use disappeared; the conditional probabilities of using any of the drugs were slightly lower in all the classes, and the prevalence of the classes showed a strong increase for No-PDU (94.7%) and a decrease for heavy-PDU (0.9%).

## 4. Discussion

### 4.1. Main Findings

This is the first study outside the USA that compares the prevalences of a large number of specific drugs between MSM and GPM, and, to our knowledge, the first ever published that compares the PDU typologies between these two populations. The study shows that prevalences are higher among MSM for all the drugs considered, with adjusted prevalence ratios, for lifetime use, ranging from one to five for the six more traditional (cannabis, hallucinogens, cocaine, ecstasy and methamphetamine), and ranging from more than ten to nearly two hundred for the four drugs that are thought to be consumed in sexual contexts among MSM populations (ketamine, GHB/GBL, volatile inhalants and mephedrone). When we focus on last-year use, these prevalence ratios at least doubled, representing greater differences in the risk of recent, and probably more regular, use than for lifetime use.

The analysis of PDU profiles shows rises in the number of drugs used and the probabilities lifetime and last-year use. The profiles in the two subpopulations are quite similar in relation to how recreational drugs get incorporated into typologies of increasing risk. However, in addition, MSM show their own typologies in each profile: beginning with poppers in the no-PDU profile and ending up incorporating all the substances associated with MSM’s sexualised drug use. Moreover, for last-year, only six out of ten MSM belonged to the lower-risk profile (No-PDU) versus nine out of ten GPM, and for lifetime, more than one in ten MSM matched the heavy-PDU profile, versus less than one in a hundred GPM.

### 4.2. Comparison with Other Studies

As can be deduced from the introduction, it is difficult to compare results from the few studies available due to the way in which drugs are grouped differently in the questionnaires or analysis. Regardless of the indicators employed, in practically all the studies mentioned and for all substances, the ratios of MSM/GPM are always above one. Data on cannabis are probably the most comparable and generally show the lowest ratio: between 1.5 and 3 in most studies and 2.3 in this study for last-year. The ratios for hallucinogens, cocaine and amphetamines tend to be very similar to that of cannabis in the two general population surveys [27,29]; however, they are higher when MSM data comes from specific MSM samples, [30] as it does with the present study. The highest prevalence ratios are always for inhalants, due to the higher poppers-use prevalence among MSM. However, once more, studies based on general-population surveys [27,29] show much lower ratios (about 4–5) than those from specific MSM samples: from 21 [30] up to 258 in the present study. We could not compare our results for the remaining drugs (greater disaggregation) with population survey studies, because they did not distinguish between some of the relevant drugs; ecstasy and ketamine are sometimes considered as “hallucinogens” [29,30]. We could only compare the prevalence ratios for “the four chems” with one other study [28]. However, in this study none of the prevalence ratios for these drugs or inhalants were higher than 5.5, while in ours the prevalence ratios were much higher—especially for mephedrone—with no last-year consumption in GPM. Comparisons of studies based on prevalence ratios between MSM and GPM are more appropriate than direct comparisons of the prevalences in MSM between studies from different countries; this is because cross-border studies will be influenced by the prevalences in the general population of the countries involved.

There are a number of different possible explanations for these differences among studies. First, convenience recruitments of MSM tend to overrepresent more risk-taking participants [45]. However, on the other hand, in general population surveys, participants tend to hide more unusual or non-normative behaviours (reporting or social desirability bias), so the use of “chemsex drugs” and/or poppers is probably more likely to be hidden than that of cannabis. As a clear example, poppers-use prevalence was much lower in all the MSM samples from general population surveys [27,29] than in those from convenience samples [28,30].

It was not possible to perform any comparison of the similarities and differences of drug-use typologies between MSM and GPM obtained through LCA in the present study, because we have not found any other studies where this analysis has also been carried out. However, as in the present study the two LCA were independent, what is feasible is to compare our MSM typologies with the few studies carried out in this subpopulation that focused on the use of drugs for any purpose. One of these [34] is not really comparable, because it targeted a 16–20-year-old population, and logically found extremely low prevalence-of-use. The study from Malaysia [33] found very low prevalence of use for all substances, with more than 80% of participants in a group who had not consumed any drug in the previous six months. This is an important difference to the present Spanish study and those from Vancouver [35] and the UK [32], because the group using chemsex drugs in Malaysia had very low probability of cannabis or poppers use. The LCA models in Vancouver, and especially in the UK—although not producing the same number of classes—show a very similar pattern of typologies: a class of minimal or practically no PDU, but who are dual users, with medium probabilities of using both cannabis and poppers; a class with preference for “conventional/old-skool” drugs; a class with preference for chemsex drugs, and a class of heavy users, with extremely high probabilities of using all kinds of drugs studied. The current study in Madrid and Barcelona found a heavy-PDU class prevalence three times higher than the other two studies, as a consequence of having the highest prevalences for all drugs considered, though MSM tend to live in big cities with higher levels of health risk behaviours.

### 4.3. Limitations

First of all, this study has employed a different way of sampling each population: probability sampling for GPM and convenience sampling for MSM. In contrast, as noted above, there are other studies where the two subpopulations were obtained using the same probability sampling. However, this approach has both advantages and disadvantages. We have already pointed out some of these when discussing possible explanations of the heterogeneity of the prevalence ratio findings from different studies. In addition, studies that use a general population survey for both GPM and MSM always have a very good sample size of GPM, but a low or modest sample of MSM. In this study, as with [30], the sizes of the two samples are more balanced. However both Woody [30] and the current study have calculated prevalence ratios between MSM and GPM that are underestimations of the MSM/heterosexual men ratios, as MSM are also included in GPM. However, the magnitude of this bias should very limited as MSM are a low percentage of GPM [37]. This bias could be compensated for by the fact that HIV positive men were not included in the MSM sample and it is known that this group present higher levels of drug use than HIV-negative MSM [13,32]. Comparing the prevalences ratios among homosexuals and heterosexuals in different countries is a more effective way to study differences than direct prevalence comparisons; this is because this approach accounts for prevalences in the corresponding general populations, which is obviously a principal contextual determinant of the prevalences in any given subpopulation.

The present study has focused on illegal substances and therefore does not present data on two important groups of psychoactive substances: “sedatives/tranquilizers/sleeping pills”, legally available on prescription but frequently obtained illegally, and “heroin or other opiates” because of the difficulties of making valid comparisons, as explained in the methods section. However, we estimate that sedative-use prevalence in MSM is double that of GPM, a figure quite similar to those found in the two studies with disaggregated data on these substances [27,30]. In the case of heroin or the main opiates we estimate that there are no differences between the two subpopulations, as also found by the only other study which makes the comparison [27].

Our study, as with all those mentioned here, did not ask about frequency of use or purpose of substance use, nor, in particular, whether substances had been consumed in sexual contexts. The findings tend to support the hypothesis that a very important component of prevalence differences could be attributable to use in sexual contexts, because the main prevalence ratio differences between the two populations were for inhalants (poppers) and “chemsex” drugs.

## 5. Conclusions

MSM showed higher prevalence ratios for having ever used all drugs for any purpose than GPM, with this difference much higher for last-year use and for those substances most considered sexualised, as inhalants and “chemsex” drugs. MSM also had different PDU-typologies characterized by the presence of these sexualised drugs in all profiles and the heavy all-drug-use profile which includes more than one in ten MSM for last-year use, rising to nearly one in five for lifetime use.

Despite the limitations discussed above on comparability due to the different sampling approach, these findings in Spain are consistent with those found in other cultural contexts, as well as with drug use studies recently carried out in MSM populations, including studies on the diffusion of chemsex. However, it is likely that the magnitude of the difference may have been overestimated. New studies that employ different MSM sampling procedures, such as convenience samples recruited online or probability surveys of the general population with questions on both sexual behaviour and drug use, are needed.

In view of these results, MSM should be considered a high priority target group when policy makers design prevention programmes for all kinds of substances and for any purpose. However, these programmes should bear in mind the heterogeneity of PDU typologies described in this population, depending on their preference either for more conventional drugs, for drugs considered as sexualised, or for both groups of substances.

In addition, new specific studies should be carried out to provide relevant information for the design of these preventive, harm reduction and care programs for physical and mental health problems appropriate to the different patterns of drug use, as well as on the role that can be played by the different types of health services where MSM who use drugs most frequently seek care: primary health care services, STI clinics or mental health services.

## Figures and Tables

**Table 1:** Comparison of sociodemographic characteristics between men who have sex with men (MSM) and general population men (GPM) in Madrid and Barcelona, 2018–2020 (%).

	MSM		GPM ^**a**^	
	*n* = 2658		*n* = 1720	
	*n*	%	*n*	%
Age group				
16–24	414	15.6 **^b^**	330	19.2
25–34	1155	43.5	470	27.3
35–44	664	25.0	466	27.1
45–54	312	11.7	268	15.6
55–64	113	4.3	186	10.8
Country of birth				
Spain	1606	60.4	1431	91.3
Latin American countries	765	28.8	29	1.8
Other countries	287	10.8	108	6.9
Size of place of residence (inhabitants)				
>1 million	2073	78.7	665	38.7
50,001–1 million	393	14.9	607	35.3
10,001–50,000	97	3.7	303	17.6
≤10,000	72	2.7	145	8.4
Education level				
≤lower secondary (≤10 education years)	169	6.4	668	39.0
Upper secondary (11–12 education years)	905	34.2	650	38.0
University (>12 education years)	1572	59.4	394	23.0
Employment status **^c^**				
Employed	1262	75.3	1134	66.9
Unemployed	120	7.2	198	11.7
Other	293	17.5	362	21.4
Cohabitation ^**c**^				
Alone	682	39.9	285	17.0
Spouse/partner without children **^d^**	359	21.0	403	24.1
Spouse/partner with children **^d^**	8	0.5	450	26.9
Children without spouse/partner **^d^**	4	0.2	33	2.0
Other relatives	270	15.8	494	29.5
Non-relatives exclusively	385	22.5	8	0.5

**^a^** All the variables showed significant differences between MSM and GPM at *p* < 0.001. **^b^** The percentages are calculated on the number of participants with known values for each variable. The proportion of missing values for the different variables was less than 3%, except for area of birth among GPM which was 8.8%. **^c^** Employment status and cohabitation among MSM were collected only in Madrid, not Barcelona. **^d^** Regardless of whether or not the participants cohabit with other relatives or non-relatives.

**Table 2:** Comparison of psychoactive substance use prevalences by last-year and lifetime use between men who have sex with men (MSM) and general-population men (GPM) in Madrid and Barcelona, 2018–2020 (%).

	Prevalence in MSM ^**a**^		Prevalence in GPM		Crude Prevalence Ratio MSM/GPM	
	Point	95% CI	Point	95% CI	Point	95% CI ^**b**^
Psychoactive Drug ^**c**^						
	Lifetime drug use					
Cannabis	57.3	55.4–59.2	47.1	44.7–49.5	1.2	1.1–1.3
Hallucinogens	10.4	9.3–11.7	7.9	6.6–9.2	1.3	1.1–1.6
Cocaine	32.9	31.2–34.8	16.7	14.9–18.5	2.0	1.8–2.2
Amphetamine	21.0	19.5–22.6	5.5	4.4–6.6	3.8	3.1–4.7
Ecstasy	31.2	29.5–33.0	6.3	5.2–7.5	5.0	4.1–6.0
Methamphetamine	16.1	14.7–17.6	2.6	1.9–3.5	6.2	4.6–8.4
Ketamine	17.7	16.3–19.2	1.2	0.7–1.8	14.7	9.5–22.9
GHB/GBL	22.3	20.8–24.0	1.2	0.7–1.8	18.6	12.0–28.8
Volatile inhalants	62.4	60.6–64.3	1.2	0.7–1.8	52.0	33.8–80.0
Mephedrone	15.1	13.8–16.6	0.1	0.0–0.3	151.2	33.4–685.3
Any drug **^d^**	77.6	76.0–79.2	48.7	46.8–50.6	1.6	1.5–1.7
	Last-year drug use					
Cannabis	40.7	38.8–42.6	17.8	15.9–19.7	2.3	2.0–2.6
Hallucinogens	4.2	3.5–5.1	1.2	0.7–1.8	3.5	2.2–5.6
Cocaine	23.6	22.1–25.3	5.1	4.1–6.3	4.6	3.7–5.7
Amphetamine	13.5	12.3–14.9	1.1	0.6–1.6	12.3	7.8–19.5
Ecstasy	23.1	21.5–24.8	1.8	1.1–2.4	12.8	9.0–18.3
Methamphetamine	12.2	11.0–13.5	0.8	0.4–1.2	15.2	8.9–26.0
Ketamine	12.4	11.2–13.8	0.2	0.0–0.5	62.1	21.3–181.2
GHB/GBL	17.7	16.3–19.2	0.1	0.0–0.4	176.8	39.4–794.1
Volatile inhalants	51.7	49.8–53.7	0.2	0.0–0.5	258.5	89.5–746.1
Mephedrone	12.4	11.2–13.7	0.0	0.0–0.2	∞	_
Any drug **^d^**	67.0	65.2–68.8	19.8	18.3–21.3	3.4	3.1–3.7
**N ^**e**^**	2658		1720			

**^a^** All the differences between the prevalences in MSM and GPM were statistically significant at *p* < 0.001, except for lifetime prevalence of hallucinogens use (*p* = 0.006). **^b^** To estimate the confidence intervals at 95% (95% CIs) of the crude prevalence ratios (cPRs), an estimate of the standard error (SE) of the natural logarithm of the prevalence ratio (lncPR) based on the normal approximation was used. SE (lncPR) ≈ $\sqrt{\left(\frac{1-p_{1}}{n_{1}p_{1}}+\frac{1-p_{2}}{n_{2}p_{2}}\right)}$, where $p_{1}$ is the prevalence in MSM, $n_{1}$ is the number of MSM in the sample, $p_{2}$ is the prevalence in GPM and $n_{2}$ is the number of GPM in the sample. **^c^** Only the most frequently used illicit psychoactive substances have been included. For this reason, alcohol, tobacco, tranquilizers/sleeping pills, opioids and other psychoactive substances used in a medical or therapeutic context have not been included. Psychoactive substances were ordered from the lowest to the highest lifetime prevalence ratios. **^d^** Use of any of the listed drugs for any purpose. **^e^** The proportion of missing values for the different substances was zero in MSM and 2% or less for GPM, except for last-year prevalence of cannabis use, which was 4.3%.

**Table 3:** Adjusted prevalence ratio (aPR) of psychoactive substance use between men who have sex with men and general-population men in Madrid and Barcelona ^**a**^, 2018–2020.

	Lifetime Drug Use		Last-Year Drug Use	
Psychoactive Drug ^**b**^	aPR	95% CI	aPR	95% CI
Cannabis	1.1	1.1–1.3	2.0	1.8–2.3
Hallucinogens	1.2	0.9–1.5	2.8	1.6–5.1
Cocaine	1.8	1.6–2.2	3.9	3.1–5.1
Amphetamine	3.6	2.9–4.7	11.3	6.7–19.0
Ecstasy	4.2	3.4–5.2	10.1	6.8–15.2
Methamphetamine	5.4	3.9–7.6	13.6	7.5–24.9
Ketamine	11.6	7.3–18.6	77.4	19.7–305.5
GHB/GBL	15.5	9.8–24.8	101.5	25.4–406.1
Volatile inhalants	53.6	33.6–85.7	234.1	75.9–722.1
Mephedrone	190.4	26.2–1388.4	_	_
Any drug **^c^**	1.5	1.4–1.6	3.1	2.8–3.5

95% CI: 95% Confidence Interval. **^a^** The results come from Poisson regression models with robust variance. Results were adjusted for age group, area of birth, province of residence (Madrid and Barcelona), size of place of residence and educational level. **^b^** Only the most frequently used illicit psychoactive substances have been included. For this reason, alcohol, tobacco, tranquilizers/sleeping pills, opioids and other psychoactive substances used in a medical or therapeutic context have not been included. Psychoactive drugs are ordered from the lowest to the highest crude prevalence ratios (See Table 2). **^c^** Use of any of the listed drugs for any purpose.

## Data Availability

The datasets used and/or analysed during the current study are available from the corresponding author on reasonable request.

## Supplementary Materials

The following are available online at https://www.mdpi.com/article/10.3390/ijerph182111609/s1, Table S1: Wording of the different psychoactive drugs included in the analysis in the two surveys, Table S2: Adjusted prevalence ratio (aPR) of psychoactive drug use between men who have sex with men and general population men in Madrid, using different sets of adjustment covariates, Table S3: Goodness-of-fit statistical information criteria comparing class membership models of polydrug use, Table S4: Results of Latent Class Analysis among men who have sex with men (MSM) and general population men (GPM) for lifetime and last-year drug use: classes, prevalence of each class and conditional probability of use of each drug within each class.

## Methysos Project Group

Marta Donat, María del Carmen Burgos and Mario González Sánchez (Instituto de Salud Carlos III, Madrid); José Antonio San Juan Bueno (Asociación Pink Peace, Madrid); Francisca Roman Urrestaruzo, Jesús E Ospina and Miguel Alarcón Gutiérrez (Agència de Salut Pública de Barcelona, Barcelona); Oskar Ayerdi, Carmen Rodríguez, Sonsoles del Corral Del Campo, Natividad Jerez Zamora, Marta Ruiz Fernández and Montserrat González Polo (Centro Sanitario Sandoval, Madrid); María Jesús Barbera Gracia, Luis López Pérez, Claudia Broto Cortes and Julio Morais Martin (CAP Drassanes, Barcelona).
