# Supplementary material for: Comparison of Polydrug Use Prevalences and Typologies between Men Who Have Sex with Men and General Population Men, in Madrid and Barcelona
Source: Int J Environ Res Public Health. 2021 Nov 4;18(21):11609. doi: 10.3390/ijerph182111609 (PMC8583212; doi:10.3390/ijerph182111609)
Supplement: Supplementary file 1 [file ijerph-18-11609-s001.zip › ijerph-1442840-supplementary.pdf]

Supplementary Table S1. Wording of the different psychoactive drugs included in the analysis in the two surveys.

Psychoactive drug	MSM[a] survey	GPM[b] survey
Cannabis	Cannabis or synthetic cannabinoids (marijuana, hashish, chocolate, joints, grass, synthetic marijuana, spice, K2)	Cannabis, marijuana or hashish (hashish oil, chocolate, pot, cost, grass)
Hallucinogens	LSD (acid, tripis)	Hallucinogens (LSD, acid, tripe, magic mushrooms, ketamine, special-K, ketolar, imalgene)
Cocaine	Powdered or crack cocaine (theme, lighthouse, base, basuco, etc.)	Powder cocaine (cocaine, parakeet, farlop, cocaine hydrochloride) or Basic cocaine (base, cooked cocaine, basuco, bowls, crack, rock)
Amphetamine	Amphetamine (speed)	Amphetamines or speed
Ecstasy	Ecstasy or MDMA in its pill form (pills, lollipops) or in its crystalline or powder form (M, crystal)	Ecstasy or other synthetic drugs (pastis, pirulas, cristal, MDMA)
Methamphetamine	Methamphetamine (bathtub, crystal meth, T)	Methamphetamine (meth, ice, crystal)
Ketamine	Ketamine (K, keta, kei)	Ketamine (also called K, Ketolar, Special K, vitamin K, k-powder)
GHB/GBL	GHB/GBL (G, canister, liquid ecstasy)	GHB [liquid ecstasy, gamma-hydroxybutyrate, X-liquid, gamma-OH]
Volatile inhalants	Poppers	Volatile inhalants (glue, glue, solvent, poppers, nitrites, gasoline)
Mephedrone	Mephedrone (mefe) or other different synthetic stimulants (bath salts, methoxetamine/MXE, methylone/3MMC, methylethcathinone/4MEC, fluoroamphetamine/light ecstasy/4FA)	Mephedrone (also called meow meow, 4-MMC, blue bubbles, catmef)

Some terms are slang names used in Spain.
[a] MSM: Men who have sex with men.
[b] GPM: General population men.

Supplementary Table S2. Adjusted prevalence ratio (aPR) of psychoactive drug use between men who have sex with men and general population men in Madrid, using different sets of adjustment covariates.

Model I[a]

Reference period	Lifetime		Last-year	
**Psychoactive drug**[c]	**aPR**	**95%CI**	**aPR**	**95%CI**
Hallucinogens	1.0	0.8-1.6	1.9	0.9-4.5
Cannabis	1.1	1.1-1.3	1.9	1.6-2.4
Cocaine	1.7	1.4-2.1	3.9	2.7-5.6
Amphetamine	3.7	2.6-5.5	9.8	4.6-21.0
Ecstasy	4.3	3.2-6.0	8.3	5.0-14.2
Methamphetamine	7.6	4.5-13.3	34.2	8.4-140.5
Ketamine	14.6	7.1-30.5	_	_
GHB/GBL	20.7	9.5-45.3	110.5	15.1-810.3
Volatile inhalants	82.4	37.4-181.6	390.7	54.6-2796.8
Mephedrone	105.0	14.4-769.3	_	_
Any drug[d]	1.6	1.5-1.7	3.2	2.7-3.7

Model II[b]

Reference period	Lifetime		Last-year	
**Psychoactive drug**[c]	**aPR**	**95%CI**	**aPR**	**95%CI**
Hallucinogens	0.9	0.6-1.4	2.0	0.8-5.1
Cannabis	1.1	1.0-1.3	1.9	1.6-2.4
Cocaine	1.4	1.2-1.8	2.9	2.0-4.4
Amphetamine	2.9	1.9-4.5	7.1	3.2-16.1
Ecstasy	3.5	2.5-5.1	6.4	3.7-11.3
Methamphetamine	5.9	3.4-10.6	21.4	5.2-88.0
Ketamine	11.1	5.1-24.1	_	_
GHB/GBL	17.0	7.4-39.2	77.8	10.5-576.8
Volatile inhalants	99.0	43.2-227.3	376.8	49.8-2854.7
Mephedrone	_	_	_	_
Any drug[d]	1.5	1.4-1.7	2.9	2.5-3.5

95% CI: Confidence Interval at 95%.

[a] The results come from Poisson regression models with robust variance. Results were adjusted for age group, area of birth, size of place of residence and education level.

[b] The results come from Poisson regression models with robust variance. Results were adjusted for age group, area of birth, size of place of residence, education level, employment status and cohabitation.

[c] Only the most frequently used illicit psychoactive substances have been included. For this reason, alcohol, tobacco, tranquilizers /sleeping pills, opioids and other psychoactive drugs used in a medical or therapeutic context have not been included. Psychoactive drugs are ordered from the lowest to the highest crude prevalence ratio (See Table 2).

[d] Use of any of the listed drug for any purpose.

Supplementary Table S3. Goodness-of-fit statistical information criteria comparing class membership models of poly-drug use.

Number of Classes	M L-LH	G2	AIC	CAIC	BIC	ABIC	Entropy
			**Lifetime drug use MSM**				
2	-10230.37	1824.415	20502.74	20626.34	20626.34	20559.61	0.9337119
3	-9813.529	990.728	19691.06	19879.4	19879.39	19777.71	0.8494151
4	-9710.974	785.6177	19507.95	19761.03	19761.02	19624.39	0.8528911
5	-9616.053	595.7767	19340.11	19657.93	19657.91	19486.34	0.7679076
6	-9600.665	564.9993	19331.33	19713.9	19713.88	19507.35	0.7760918
			**Last-year drug use MSM**				
2	-9153.162	1425.354	18348.32	18471.92	18471.92	18405.19	0.9324011
3	-8862.53	844.0906	17789.06	17977.4	17977.39	17875.72	0.8308068
4	-8775.847	670.7237	17637.69	17890.78	17890.76	17754.14	0.8451024
5	-8729.919	578.869	17567.84	17885.67	17885.65	17714.07	0.7626853
			**Lifetime drug use GPM**				
2	-2694.337	378.6877	5430.673	5544.606	5544.593	5477.879	0.9679066
3	-2565.937	121.8892	5195.875	5369.486	5369.467	5267.808	0.8917339
4	-2541.913	73.8405	5169.826	5403.116	5403.091	5266.486	0.8888671
5	-2535.093	60.20011	5178.186	5471.155	5471.123	5299.572	0.8989366
			**Last-year drug use GPM**				
2	-1255.291	116.7728	2548.583	2650.867	2650.855	2590.495	0.9642639
3	-1231.596	69.38156	2521.191	2677.309	2677.291	2585.164	0.9559468
4	-1222.294	50.77898	2522.589	2732.54	2732.516	2608.62	0.9623893

Supplementary Table S4. Results of Latent Class Analysis among men who have sex with men (MSM) and general population men (GPM) for lifetime and last-year drug use: classes, prevalence of each class and conditional probability of use of each drug within each class

	MSM				GPM			
**Latent Class**	Class 1: No-PDU[a]	Class 2: Conventional PDU plus poppers	Class 3: PDU preferring chemsex drugs	Class 4: Heavy PDU	Class 1: No-PDU	Class 2: Conventional PDU	Class 3: Intensive conventional PDU	Class 4: Heavy PDU
				**Lifetime drug use**				
**Probability of drug use**								
Cannabis	0.38	0.89	0.44	0.92	0.34	1.00	0.96	1.00
Hallucinogens	0.00	0.14	0.00	0.45	0.00	0.16	0.77	1.00
Cocaine	0.03	0.64	0.46	0.94	0.01	0.63	1.00	0.97
Amphetamine	0.00	0.27	0.14	0.88	0.00	0.00	0.75	1.00
Ecstasy	0.02	0.61	0.26	0.97	0.00	0.09	0.68	1.00
Methamphetamine	0.00	0.12	0.32	0.68	0.00	0.02	0.24	0.68
Ketamine	0.00	0.10	0.20	0.84	0.00	0.01	0.00	0.61
GHB/GBL	0.00	0.18	0.61	0.86	0.00	0.00	0.07	0.48
Volatile inhalants	0.41	0.85	0.95	0.98	0.00	0.02	0.07	0.34
Mephedrone	0.00	0.00	0.52	0.69	0.00	0.00	0.00	0.03
**Class prevalence**	57.7	18.8	6.4	17.2	79.6	13.8	4.9	1.8
				**Last-year drug use**				
**Latent Class**	Class 1: No-PDU	Class 2: Conventional PDU plus poppers	Class 3: PDU preferring chemsex drugs	Class 4: Heavy PDU	Class 1: No-PDU	Class 2: Conventional PDU		Class 3: Heavy PDU
**Probability of drug use**								
Cannabis	0.25	0.76	0.26	0.80	0.14	0.86		0.92
Hallucinogens	0.00	0.06	0.00	0.24	0.00	0.15		0.64
Cocaine	0.02	0.52	0.37	0.89	0.01	0.75		0.79
Amphetamine	0.00	0.17	0.14	0.73	0.00	0.04		0.74
Ecstasy	0.01	0.54	0.18	0.93	0.00	0.19		0.92
Methamphetamine	0.00	0.11	0.28	0.64	0.00	0.06		0.50
Ketamine	0.00	0.09	0.12	0.75	0.00	0.00		0.13
GHB/GBL	0.00	0.14	0.61	0.85	0.00	0.00		0.13
Volatile inhalants	0.33	0.75	0.90	0.96	0.00	0.01		0.20
Mephedrone[b]	0.00	0.00	0.51	0.68	_	_		_
**Class prevalence**	64.7	15.6	6.2	13.5	94.7	4.4		0.9

[a] PDU: Polidrug users
[b] Mephedrone is not displayed because there were not users in the last 12 months among GPM.
